# HOXB13 overexpression is an independent predictor of early PSA recurrence in prostate cancer treated by radical prostatectomy

**DOI:** 10.18632/oncotarget.3431

**Published:** 2015-03-23

**Authors:** Cristina Villares Zabalza, Meike Adam, Christoph Burdelski, Waldemar Wilczak, Corina Wittmer, Stefan Kraft, Till Krech, Stefan Steurer, Christina Koop, Claudia Hube-Magg, Markus Graefen, Hans Heinzer, Sarah Minner, Ronald Simon, Guido Sauter, Thorsten Schlomm, Maria Christina Tsourlakis

**Affiliations:** ^1^ Institute of Pathology, University Medical Center Hamburg-Eppendorf, Germany; ^2^ Martini-Clinic, Prostate Cancer Center, University Medical Center Hamburg-Eppendorf, Germany; ^3^ General, Visceral and Thoracic Surgery Department and Clinic, University Medical Center Hamburg-Eppendorf, Germany; ^4^ Department of Urology, Section for Translational Prostate Cancer Research, University Medical Center Hamburg-Eppendorf, Germany

**Keywords:** HOXB13, ERG, PTEN, TMA, prostate cancer

## Abstract

HOXB13 is a prostate cancer susceptibility gene which shows a cancer predisposing (G84E) mutation in 0.1–0.6% of males. We analyzed the prognostic impact of HOXB13 expression by immunohistochemistry on a tissue microarray containing more than 12,400 prostate cancers. Results were compared to tumor phenotype, biochemical recurrence, androgen receptor (AR) and prostate specific antigen (PSA) as well as molecular subtypes defined by ERG status and genomic deletions of 3p, 5q, 6q, and PTEN. HOXB13 immunostaining was detectable in 51.7% of 10,216 interpretable cancers and considered strong in 9.6%, moderate in 19.7% and weak in 22.3% of cases. HOXB13 expression was linked to advanced pT stage, high Gleason grade, positive lymph node status (*p* < 0.0001 each), high pre-operative PSA levels (*p* = 0.01), TMPRSS2:ERG fusion, PTEN deletions, AR expression, cell proliferation, reduced PSA expression and early PSA recurrence (*p* < 0.0001 each). The prognostic value of HOXB13 was independent from established parameters including Gleason, stage, nodal stage and PSA. Co-expression analysis identified a subset of tumors with high HOXB13 and AR but low PSA expression that had a particularly poor prognosis. HOXB13 appears to be a promising candidate for clinical routine tests either alone or in combination with other markers, including AR and PSA.

## INTRODUCTION

Prostate cancer is the most prevalent cancer in men in Western societies [1]. Although most prostate cancers have a rather indolent clinical course, prostate cancer represents the third most common cause of cancer related death in men. Despite recent advances in molecular research, the only established pretreatment prognostic parameters include Gleason grade and tumor extent on biopsies, preoperative prostate-specific antigen (PSA), and clinical stage. Because these data are statistically powerful but not sufficient for optimal individual treatment decisions, it can be hoped that a better understanding of disease biology will eventually lead to the identification of clinically applicable molecular markers that enable a more reliable prediction of prostate cancer aggressiveness.

The homeobox (HOX) is a specific and widely distributed DNA sequence characterizing genes that are responsive to HOX transcription factors [2]. HOX transcription factors are highly conserved major regulators of developmental processes in many organ systems. Homeobox B13 (HOXB13) is one of the numerous DNA-binding transcription factors of the HOX gene family that has been implicated in the development of normal prostate and cancer [3]. In the normal prostate gland, HOXB13 forms complexes with the androgen receptor (AR) and acts as an important modulator of the transcriptional activity of both androgen independent and androgen responsive genes [4]. Depending on the presence or absence of other transcription factors these complexes can stimulate transcription of certain target genes and suppress others – such as prostate specific antigen (PSA) [4]. Recent data have established HOXB13 as a strong candidate gene for causing hereditary prostate cancer if mutated. A rare HOXB13 polymorphism (G84E) occurring in 0.1–0.6% of European/American populations has been linked to an increased prostate cancer risk by several groups [5–10]. Men with G84E mutations have a 3–5 fold higher risk for developing prostate cancer and develop their tumors earlier than patients without this deletion [6, 11]. Several studies have further used immunohistochemistry to show that HOXB13 is often expressed at high levels in prostate cancer [12–15].

Given the potential importance of HOXB13 for prostate cancer development, we wondered whether HOXB13 expression levels would be linked to disease outcome or relevant clinical or molecular subgroups. For this purpose, we took advantage of our preexisting tissue microarray containing more than 12,400 prostate cancer specimens with clinical follow-up and attached molecular database. The results of our study demonstrate that high levels of HOXB13 is tightly linked to unfavorable patient outcome.

## RESULTS

### Technical issues

A total of 10,216 (91.6%) of tumor samples were interpretable in our TMA analysis. Reason for non-informative cases (935 spots; 8.4%) included lack of tissue samples or absence of unequivocal cancer tissue in the TMA spot.

### HOXB13 immunohistochemistry

**Figure 1:**
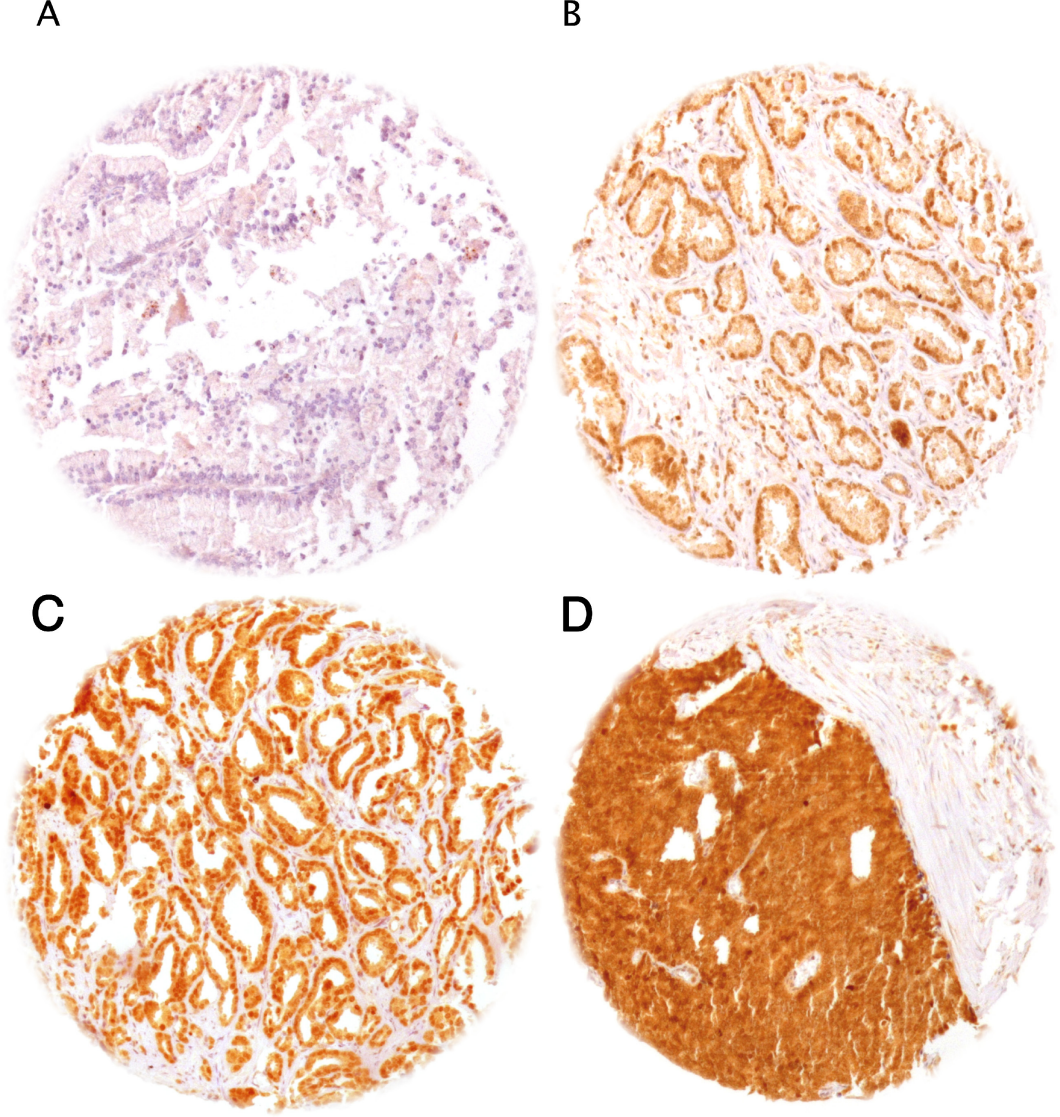
Representative pictures of HOXB13 immunostaining in prostate cancer **(A)** negative (no nuclear staining), **(B)** weak, **(C)** moderate, and **(D)** strong staining.

Normal prostatic glands showed weak nuclear staining. Positive staining was limited to the secretory epithelial cells, while basal cells were consistently negative. In cancers, HOXB13 staining was seen in 5,278 of our 10,216 (51.7%) interpretable tumors and was considered weak in 22.3%, moderate in 19.7% and strong in 9.6% of cancers (Table 1). Representative images of HOXB13 immunostainings are given in Figure 1A–1D.

**Table 1:** Association between HOXB13 immunostaining results and prostate cancer phenotype

	*n* evaluable	HOXB13 IHC result (%)				*P* value
		negative	weak	moderate	strong	
**All cancers**	10, 216	48.3	22.3	19.7	9.6	
**Tumor stage**						
pT2	6, 691	52.1	22.6	17.8	7.4	< 0.0001
pT3a	2, 241	43.1	21.7	22.5	12.8	
pT3b	1, 177	36.8	22.3	24.8	16.1	
pT4	62	37.1	14.5	33.9	14.5	
**Gleason grade**						
≤ 3 + 3	2, 577	60.3	20.1	15.0	4.6	< 0.0001
3 + 4	5, 693	46.3	23.6	20.4	9.7	
4 + 3	1, 448	37.7	21.6	25.1	15.5	
≥ 4 + 4	442	37.6	21.5	22.2	18.8	
**Lymph node metastasis**						
N0	5, 657	46.2	22.6	20.1	11.1	< 0.0001
N+	528	34.7	20.9	24.6	19.9	
**Preop. PSA level (ng/ml)**						
< 4	1, 267	46.3	22.6	22.1	9.0	0.01
4–10	6, 137	48.0	23.3	19.3	9.4	
> 10–20	2, 006	48.7	21.0	19.7	10.5	
> 20	686	52.2	17.8	10.5	10.6	
**Surgical margin**						
negative	8, 212	48.7	22.8	19.2	9.4	0.0004
positive	1, 824	46.6	20.1	22.5	10.9	

### Association with *TMPRSS2:ERG* fusion status and ERG protein expression

**Figure 2:**
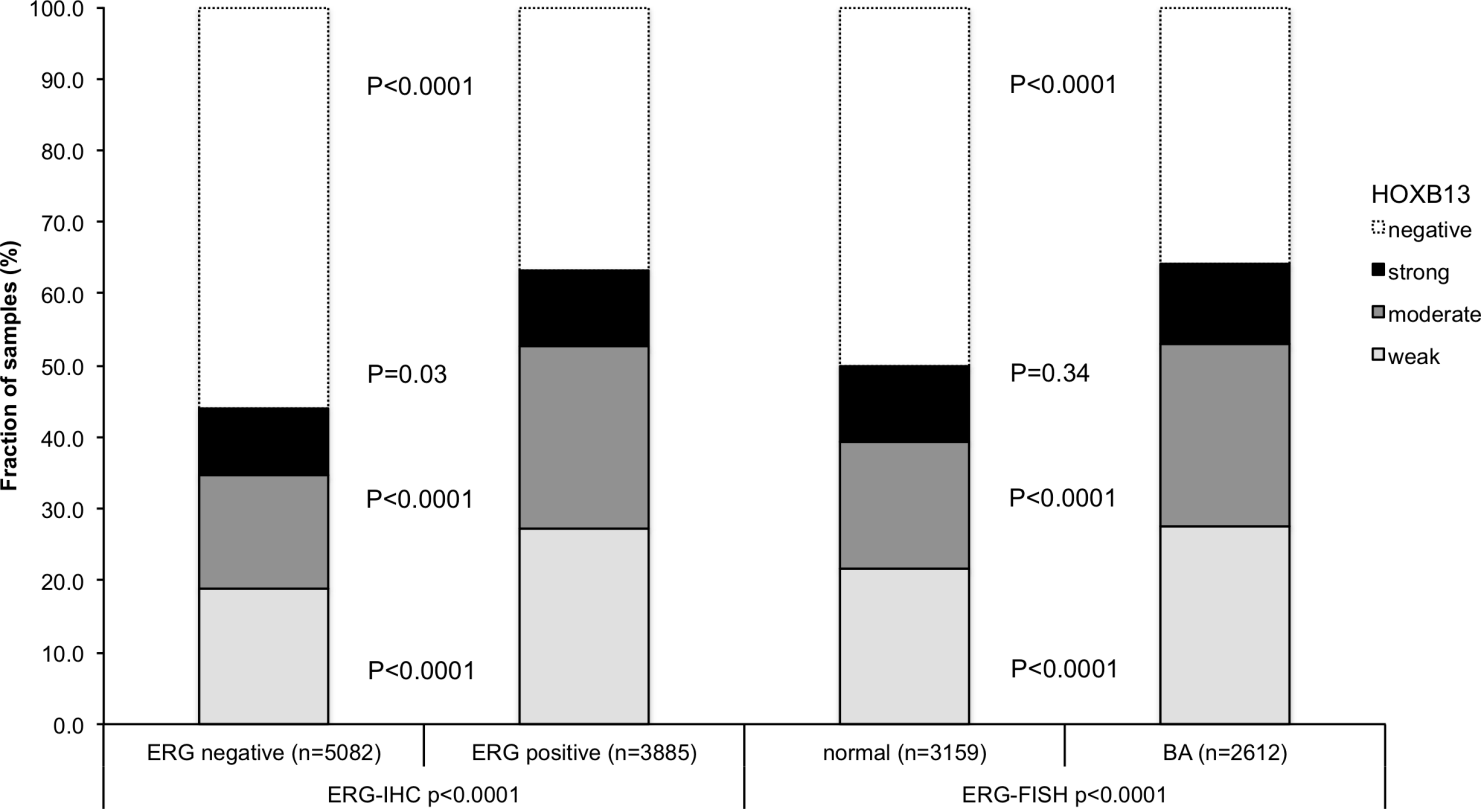
Association between HOXB13 immunostaining results and the ERG-status determined by IHC and FISH analysis BA (break apart) indicates rearrangement of the ERG gene according to FISH analysis.

To evaluate whether HOXB13 expression is associated with ERG status in prostate cancers, we used data from previous studies (expanded from [18, 19]). Data on *TMPRSS2:ERG* fusion status obtained by FISH were available from 5,677 and by immunohistochemistry from 8,459 tumors with evaluable HOXB13 immunostaining. Data on both ERG FISH and IHC were available from 5,468 cancers, and an identical result (ERG IHC positive and break by FISH or ERG IHC negative and missing break by FISH) was found in 5,231 of 5,468 (95.7%) cancers. HOXB13 immunostaining was strongly linked to presence of *TMPRSS2:ERG* rearrangements and ERG expression. HOXB13 expression was seen in 63.4% (ERG IHC) and 64.1% (ERG FISH) of ERG-positive cancers but in only 44.1% and 49.9% of cancers without ERG staining and ERG rearrangement, respectively (*p* < 0.0001 each; Figure 2). HOXB13 immunostaining was linked to advanced pathological tumor stage, high Gleason grade, and lymph node metastasis (*p* < 0.0001 each) in subsets of both ERG-negative (Supplementary Table 2) and ERG-positive cancers (Supplementary Table 3).

### Association to key genomic deletions

**Figure 3:**
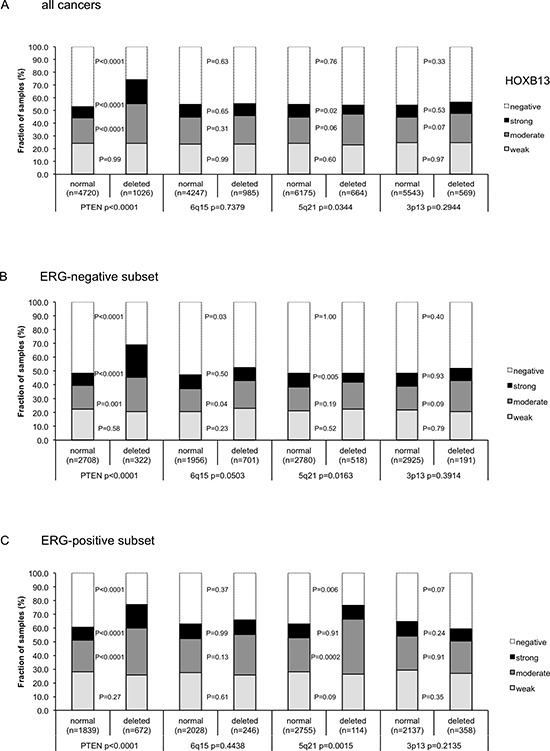
Association between positive HOXB13 immunostaining results and deletions of PTEN, 5q21 (CHD1), 6q15 (MAP3K7), and 3p13 (FOXP1) **(A)** all cancers, **(B)** ERG-negative and **(C)** ERG-positive subset according to ERG-IHC analysis.

Earlier studies had provided evidence for recurrent chromosomal deletions delineating further molecular subgroups amongst ERG positive and ERG negative prostate cancers. In particular, deletions of *PTEN* and 3p13 define subgroups in ERG positive and deletions of 5q21 and 6q15 define subgroups in ERG negative cancers [20, 21, 23]. To examine, whether HOXB13 expression might be particularly associated with one of these genomic deletions, HOXB13 data were compared to preexisting findings on *PTEN* (10q23), 3p13 (*FOXP1*), 6q15 (*MAP3K7*) and 5q21(*CHD1*) deletions. Elevated HOXB13 expression levels were strongly linked to deletions of PTEN in all cancers (Figure 3A) as well as in the subsets of ERG- negative (Figure 3B) and ERG- positive cancers (*p* < 0.0001 each, Figure 3C). However, HOXB13 was largely unrelated to all other deletions irrespective of whether all cancers or subgroups of ERG positive or ERG negative cancers were analyzed.

### Associations between immunohistochemical expression of HOXB13, AR and PSA

**Figure 4:**
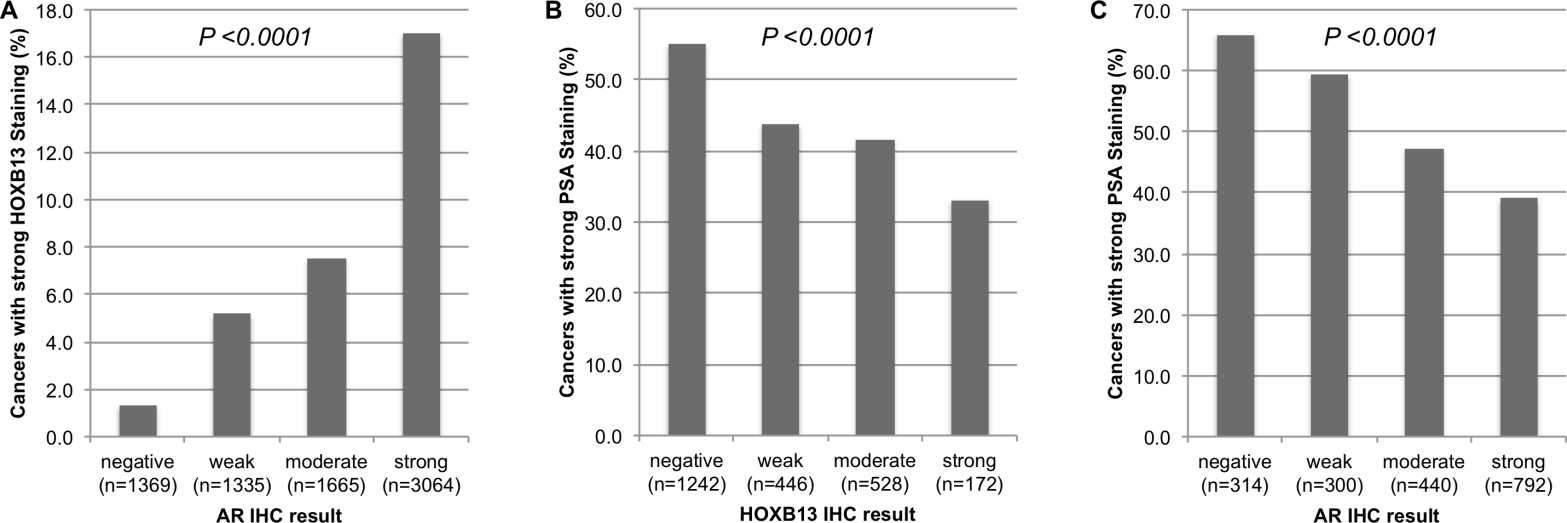
Correlation of IHC parameters HOXB13, androgen receptor (AR) and cytoplasmic PSA **(A)** strong HOXB13 expression and AR, **(B)** strong PSA expression and HOXB13, and **(C)** strong PSA expression and AR.

To search for associations between expression of HOXB13 and its co-regulator AR, we included data from a previous study [19]. There was a strong positive link between overexpression of HOXB13 and high-level AR expression (*p* < 0.0001, Figure 4A). Strong HOXB13 expression was found in 17% of cancers with strong AR expression, but only in 1.3% of AR-negative tumors. HOXB13 and AR expression were further compared to PSA immunohistochemical results because PSA was described to be down regulated by AR/HOXB13 [25]. As expected from these studies, HOXB13 was inversely linked to PSA (*p* < 0.0001, Figure 4B). Strong PSA expression was seen in 55.1% of HOXB13-negative tumors, but only in 33.1% of cancers with strong HOXB13 expression. Likewise, AR expression was inversely related to PSA levels. High-level PSA was found in 65.9% of AR-negative cancers, but only in 39% of strongly AR-positive tumors (*p* < 0.0001, Figure 4C).

### Association to tumor cell proliferation (Ki67LI)

Strong HOXB13 staining was significantly linked to accelerated cell proliferation as measured by Ki67LI in all cancers (*p* < 0.0001). This association held also true with high significance in most subgroups of cancers with comparable Gleason grade (≤ 3 + 3; 3 + 4; 4 + 3; ≥4 + 4, Table 2).

**Table 2:** Associations between HOX13B immunohistochemistry results and cell proliferation as measured by Ki67 immunohistochemistry in all cancers and subsets of cancers defined by Gleason grade

		HOXB13 IHC	*n*	Ki67LI		*P* value
**All cancers**		negative	3,225	2.28	±0.05	< 0.0001
		weak	1,519	3.09	±0.07	
		moderate	1,309	3.14	±0.07	
		strong	658	3.51	±0.10	
**Gleason**
	≤ 3 + 3	negative	936	1.89	±0.07	< 0.0001
		weak	312	2.67	±0.12	
		moderate	219	2.40	±0.14	
		strong	73	2.77	±0.24	
	3 + 4	negative	1,798	2.26	±0.06	< 0.0001
		weak	944	2.89	±0.08	
		moderate	782	3.10	±0.08	
		strong	381	3.28	±0.12	
	4 + 3	negative	372	3.00	±0.18	0.003
		weak	197	4.04	±0.25	
		moderate	236	3.48	±0.22	
		strong	149	3.83	±0.28	
	≥ 4 + 4	negative	95	3.76	±0.49	0.15
		weak	62	5.29	±0.60	
		moderate	68	4.88	±0.58	
		strong	52	5.23	±0.66	

Abbreviations: Ki67LI = Ki67 labeling index (mean±SEM).

### Association with PSA recurrence

**Figure 5:**
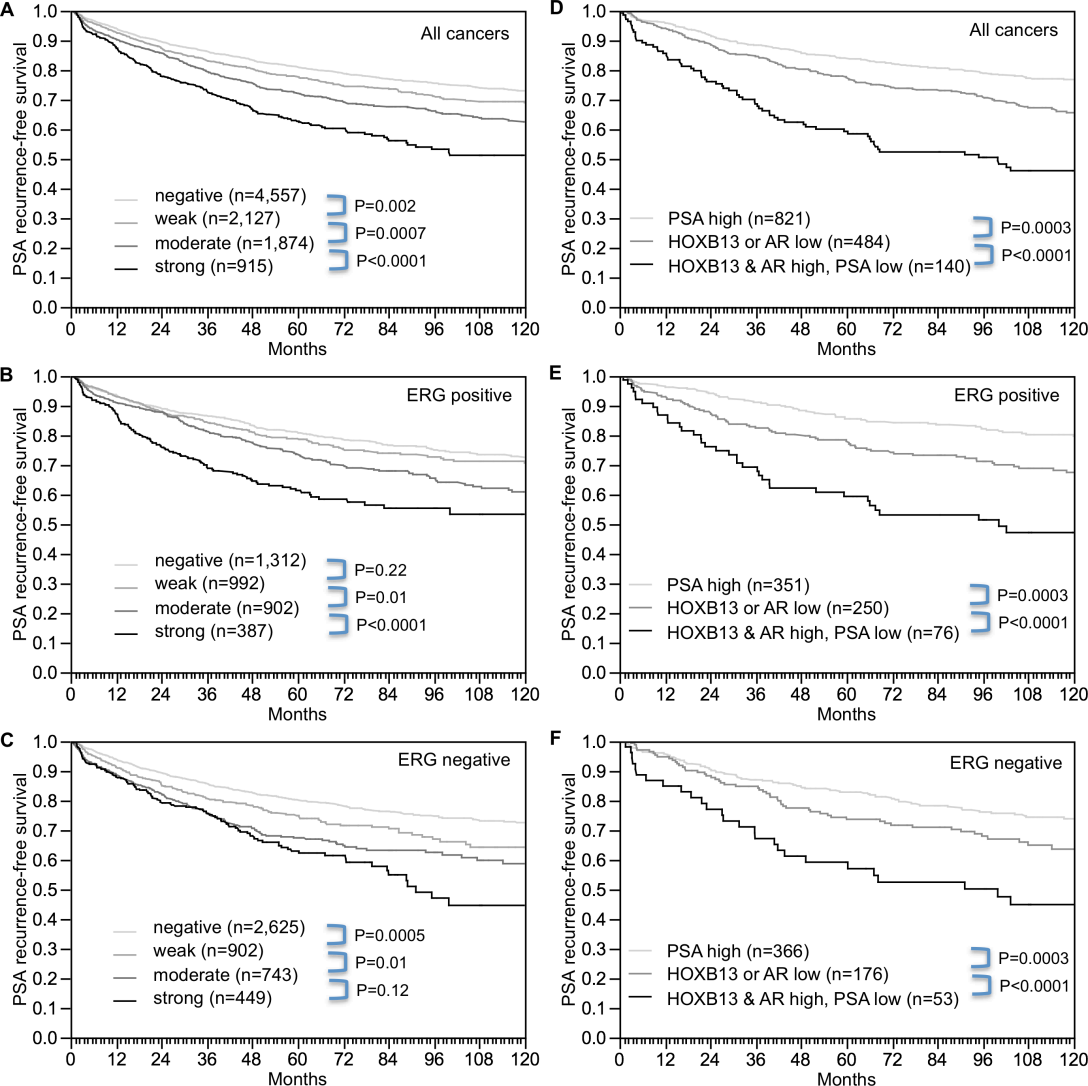
Association between HOXB13 expression and biochemical recurrence **(A)** all cancers, **(B)** ERG-IHC positive cancers, and **(C)** ERG-IHC negative cancers. Combined effect of HOXB13, AR and PSA in **(D)** all cancers, **(E)** ERG-IHC positive cancers, and **(F)** ERG-IHC negative cancers.

Follow-up data were available from 9,474 patients with interpretable HOXB13 immunostaining on the TMA. Increasing levels of HOXB13 were equally paralleled by decreasing PSA recurrence-free intervals if all cancers were jointly analyzed (*p* < 0.0001, Figure 5A), as in subsets of ERG-IHC-positive (*p* < 0.0001, Figure 5B) or ERG-IHC-negative cancers *p* < 0.0001, Figure 5C). Because of the strong associations between HOXB13, AR and PSA, we extended the analyses to tumor subgroups stratified according to the following criteria 1) cancers with strong PSA staining irrespective of the HOXB13 and AR results (group 1: PSA high), 2) cancers with low (negative to weak) HOXB13 expression or low (negative to moderate) AR expression but low (negative-moderate) PSA expression (group 2: HOXB13 or AR low), and cancers with high (moderate-strong) HOXB13 expression and high (strong) AR expression but low PSA expression (group 3: HOXB13 and AR high, PSA low). These analyses revealed marked prognostic differences: Tumors with high PSA had the best, and tumors with high HOXB13 and AR, but low PSA, had the worst prognosis, while an intermediate prognosis was found for tumors with low expression of at least one of HOXB13 or AR (*p* < 0.0001, Figure 5D). These differences held also true in subsets of ERG-positive (*p* < 0.0001, Figure 5E) and ERG-negative cancers (*p* < 0.0001, Figure 5F).

### Multivariate analysis

Four different types of multivariate analyses were performed evaluating the clinical relevance of HOXB13 expression in different scenarios (Table 3). Scenario 1 evaluated all postoperatively available parameters including pathological tumor stage, pathological lymph node status (pN), surgical margin status, preoperative PSA value and pathological Gleason grade obtained after the morphological evaluation of the entire resected prostate. In scenario 2, all postoperatively available parameters with exception of nodal status were included. The rational for this approach was that the indication and extent of lymph node dissection is not standardized in the surgical therapy of prostate cancer and that excluding pN in multivariate analysis can markedly increase case numbers. Two additional scenarios had the purpose to model the preoperative situation as much as possible. Scenario 3 included HOXB13 expression, preoperative PSA, clinical tumor stage (cT stage) and Gleason grade obtained on the prostatectomy specimen. Since postoperative determination of a tumors Gleason grade is “better” than the preoperatively determined Gleason grade (subjected to sampling errors and consequently under-grading in more than one third of cases [26]), another multivariate analysis was added. In scenario 4, the preoperative Gleason grade obtained on the original biopsy was combined with preoperative PSA, cT stage and HOXB13 expression. These analyses revealed that HOXB13 expression levels provided independent prognostic information in almost all scenarios.

**Table 3:** Multivariate analysis including HOXB13 expression in all cancers, the ERG-negative and ERG-positive subset Scenario 1 includes all postoperatively parameters. Scenario 2 excludes the lymph node (pN) stage. Scenario 3 replaces the pathological tumor (pT) stage with the clinical (cT) stage and omits the surgical margin (R) status. Scenario 4 includes all preoperative available parameters

Tumor subset	Scenario	*n* analyzable	*P* value							
			preop. PSA-Level	pT Stage	cT Stage	Gleason grade pros tatectomy	Gleason grade biopsy	pN Stage	R-Status	HOXB13-Expression
**all cancers**	1	5,614	< 0.0001	< 0.0001	-	< 0.0001	-	< 0.0001	< 0.0001	0.005
	2	9,261	< 0.0001	< 0.0001	-	< 0.0001	-	-	< 0.0001	< 0.0001
	3	9,121	< 0.0001	-	< 0.0001	< 0.0001	-	-	-	< 0.0001
	4	8,990	< 0.0001	-	< 0.0001	-	< 0.0001	-	-	< 0.0001
**ERG-negative cancers**	1	2,871	< 0.0001	< 0.0001	-	< 0.0001	-	< 0.0001	0.27	0.02
	2	4,616	< 0.0001	< 0.0001	-	< 0.0001	-	-	0.004	0.002
	3	4,577	< 0.0001	-	< 0.0001	< 0.0001	-	-	-	0.0002
	4	4,517	< 0.0001	-	< 0.0001	-	< 0.0001	-	-	< 0.0001
**ERG-positive cancers**	1	2,170	0.009	< 0.0001	-	< 0.0001	-	0.17	0.0003	0.11
	2	3,511	0.0002	< 0.0001	-	< 0.0001	-	-	< 0.0001	0.06
	3	3,431	< 0.0001	-	< 0.0001	< 0.0001	-	-	-	0.006
	4	3,375	< 0.0001	-	< 0.0001	-	< 0.0001	-	-	0.0002

## DISCUSSION

The results of our study identify HOXB13 overexpression as a strong prognostic factor in prostate cancer, and suggest clinically relevant in-vivo interactions with its binding partner androgen receptor.

The immunohistochemistry protocol was designed to detect expression differences between individual prostate cancers in this study. At the selected conditions, our analysis of 10,216 patients revealed no staining in 48.3% of tumors while 22.3% had a weak and 29.3% a moderate to strong HOXB13 immunostaining. These findings indeed suggest a substantial variability of HOXB13 expression in prostate cancer. Data from two other studies using a more sensitive IHC protocol had earlier demonstrated that at least some HOXB13 expression can occur in every prostate cancer. Varinot et al. described strong HOXB13 immunostaining in all 400 analyzed prostate cancers and in secretory cells from all analyzed 120 normal prostates [13], while in another study, moderate to strong HOXB13 expression was found in only 30% of 44 prostate cancers [12]. These discrepant findings on the HOXB13 positivity rate further illustrate the paramount impact of protocols on IHC data. In an earlier study, we described IHC protocol induced variability of p53 positivity between < 5 and > 95% [27]. The significant associations of HOXB13 staining levels with various clinical, pathological and molecular tumor parameters indicate, that our measurements reflect true and biologically relevant expression differences between cancers. The data also demonstrate, that prostate cancers generally show increased HOXB13 expression levels as compared to normal prostate epithelium. This latter observation is in line with a role of HOXB13 overexpression during malignant transformation of prostate epithelial cells.

The data from this study further corroborate recently suggested interactions between HOXB13, AR, and PSA. Work by several groups has indicated, that HOXB13 and androgen receptor complex with each other, and that these complexes jointly regulate the expression of androgen dependent genes [4, 25, 28]. The strong positive correlation of the expression levels of HOXB13 and AR as measured on our TMA is consistent with these assumptions derived from xenograft and cell line experiments [28]. The strong inverse association of HOXB13 and AR expression with PSA staining levels of our tumors represents a strong *in vivo* validation for down regulation of PSA by AR/HOXB13 that was earlier assumed from cell line experiments [4]. That high-level PSA can be found in the majority of AR-negative cancers can be explained by AR-independent up regulation of PSA by the mitogen activated protein (MAP) kinase pathway [29]. Importantly, finding at least some inverse associations of biomarkers always provides strong indirect evidence for the validity of our IHC data. It must be assumed, that a small number (< 10%) of non-reactive or “under reactive” tissues are regularly included in molecular studies using routinely processed patient material. An unusually high rate of such cases could thus induce a “false” positive association of biomarkers due to concordant negative or “low expression” results for all evaluated biomarkers in a distinct “non-reactive” subgroup of cancers. Inverse associations cannot be caused by a proportion of non-reactive tissues yielding negative IHC results for all analyzed antibodies.

Our notion, that HOXB13 is highly relevant for the biology of prostate cancer is further substantiated by the strong link of high expression with unfavorable tumor phenotype and poor outcome in this study. The high number of cancers included in this analysis further enabled us to study the combined impact of the interrelated biomarkers HOXB13, AR, and PSA. These data suggest a markedly unfavorable prognosis in patients with tumors showing highest evidence for a strong activity of the HOXB13/AR complex including a measurable PSA down-regulation. The strong and independent prognostic impact of this combined feature is in line with the critical role of androgen receptor signaling in prostate cells.

The abundant molecular database attached to our tumor collection further enabled us to analyze the role of HOXB13 in tumors of different molecular subtypes, the most common of which is the TMPRSS2:ERG gene fusion. Gene fusions involving the androgen-regulated gene TMPRSS2 and ERG occur in about 50% of prostate cancers and result in strong ERG protein overexpression [30] with consequent deregulation of the expression of a large number of genes [31–34]. The strong association of HOXB13 with positive ERG status expression fits well with the known role of androgen receptor activation for development of ERG fusions in prostate cancer [35]. Experimental studies have demonstrated that ERG fusions develop as a result of elevated androgen signaling [36]. About 0.1–0.6% of men carry a specific HOXB13 germ line mutation (G84E) [5–10, 37]. Interestingly, it was recently shown, that only 5 of 23 (21.7%) analyzed prostate cancers from patients with a G84E mutation had an ERG fusion [38] as compared to about 50% in unselected cohorts [30]. This observation would be consistent with models suggesting that germ line G84E mutations of HOXB13 negatively impact androgen signaling to an extent that ERG fusion development are less likely to occur.

Next to ERG fusions, chromosomal deletions represent the most common recurrent genomic alterations in prostate cancer. Increasing amount of data is suggesting that these deletions may delineate biologically and clinically relevant subgroups within ERG positive and ERG negative cancers as most of these deletions are clearly linked to ERG status. Deletions at 3p13 and 10q23 (PTEN) are associated with ERG-positive cancers, while 5q21 and 6q15 are inked to ERG-negative cancers [22, 39–41]. That several biomarkers considered relevant for prostate cancer cluster with some of these deletions further supports the potential significance of molecular prostate cancer subgroups defined by deletions. For example, CRISP3 overexpression is strongly associated with PTEN deleted ERG positive prostate cancer [42]. SPINK1 expression is tightly linked to 6q15- and 5q21-deleted ERG negative prostate cancers [43]. The data from this study revealed a striking link of strong HOXB13 expression with PTEN deleted ERG positive cancers. Since HOXB13 is involved in both positive and negative growth control through activation of AKT/mTOR signaling [44] and inhibition of CCND1 [14], it is possible that the association between HOXB13 expression and PTEN deletion reflects compensatory up regulation of HOXB13 in cancer cells with severely deregulated AKT signaling.

Among all cancers, prostate cancer is the one tumor where predictors of the natural course of the disease are most needed. The majority of men develop prostate cancer during their lifetime but only relatively few of them will eventually die of their tumor [45, 46]. Accidental diagnosis of a low-grade prostate cancer thus poses the problem whether or not this cancer needs therapy. Many individual parameters have been demonstrated as potentially prognostic in earlier studies, but results on most parameters are conflicting [47]. Recent studies have shown promising data for multiparametric prognostic tests in prostate cancer [48, 49] and several tests are now commercially available to patients [50–52]. It is most likely, however, that better tests can be developed. For example, several independent prognostic parameters have been identified by our group by using the same large-scale TMA approach as in this study [49, 53–55]. It appears likely, that several of these features as well as other parameters that remain to be detected could add substantially to existing multiparametric tests. Given the strong and independent prognostic role for high HOXB13 expression in all models, we thus hypothesize, that this parameter will be applicable for routine evaluation of prostate cancers – most likely in combination with other relevant features, such as for example AR and PSA expression levels.

In summary, our study highlights HOXB13 as strong and independent prognostic marker in prostate cancer, and a promising candidate for a routine clinical application.

## MATERIALS AND METHODS

### Patients

Radical prostatectomy specimens were available from 11,152 patients, undergoing surgery between 1992 and 2011 at the Department of Urology and the Martini Clinics at the University Medical Center Hamburg-Eppendorf. Follow-up data were available for a total of 7,440 patients with a median follow-up of 36 months (range: 1 to 228 months; Supplementary Table 1). Prostate specific antigen (PSA) values were measured following surgery and PSA recurrence was defined as a postoperative PSA of 0.2 ng/ml and increasing at first of appearance. All prostate specimens were analyzed according to a standard procedure, including a complete embedding of the entire prostate for histological analysis [16]. The TMA manufacturing process was described earlier in detail [17]. In short, one 0.6mm core was taken from a representative tissue block from each patient. The tissues were distributed among 27 TMA blocks, each containing 144 to 522 tumor samples. For internal controls, each TMA block also contained various control tissues, including normal prostate tissue. The molecular database attached to this TMA contained results on ERG expression in 10,711 [18], ERG break apart FISH analysis in 7,122 (expanded from [19]) and deletion status of 5q21 (CHD1) in 7,932 (expanded from [20]), 6q15 (MAP3K7) in 6,069 (expanded from [21]), PTEN (10q23) in 6,704 (expanded from [22]), and 3p13 (FOXP1) in 7,081 (expanded from [23]), Further immunohistochemical data were available on Ki67LI from 7,010 [24], and on androgen receptor (AR) in 7,856 tumors. (expanded from [24]).

### Immunohistochemistry

Freshly cut TMA sections were immunostained on one day and in one experiment. For HOXB13 immunohistochemistry, slides were deparaffinized and exposed to heat-induced antigen retrieval for 5 minutes in an autoclave at 121°C in pH 7.8 Tris-EDTA-Citrate buffer. Primary antibody specific for HOXB13 (mouse monoclonal antibody, Abcam, Cambridge, UK; cat# 53931; dilution 1:25) was applied at 37°C for 60 minutes. These conditions were selected in order to optimally distinguish between different expression levels of HOXB13. At a dilution of 1:25, there were both strongly nuclear positive but also entirely negative prostate cancers. HOXB13 staining was predominantly nuclear and typically paralleled by cytoplasmic co-staining. For PSA immunohistochemistry, slides were deparaffinized. No antigen retrieval step was performed. Primary antibody specific for PSA (mouse monoclonal antibody, Novocastra; cat# NCL-L-PSA-28A4; dilution 1:50) was applied at 37°C for 60 minutes. PSA staining was localized to the cytoplasm. Bound HOXB13 and PSA antibodies were visualized using the EnVision Kit (Dako, Glostrup, Denmark) according to the manufacturer´s directions. Nuclear HOXB13 staining was evaluated according to the following scoring system: The staining intensity (0, 1+, 2+, and 3+) and the fraction of positive tumor cells were recorded for each tissue spot. A final score was built from these two parameters according to the following established criteria. Negative: absence of staining; weak: staining intensity of 1+ in ≤ 70% of tumor cells or staining intensity of 2+ in ≤ 30% of tumor cells; moderate: staining intensity of 1+ in > 70% of tumor cells or staining intensity of 2+ in > 30% but in ≤ 70% of tumor cells or staining intensity of 3+ in ≤ 30% of tumor cells; strong: staining intensity of 2+ in > 70% of tumor cells or staining intensity of 3+ in > 30% of tumor cells. Cytoplasmic PSA staining intensity was estimated in a four-step scale including negative (no detectable staining), weak, moderate, and strong staining.

### Statistics

For statistical analysis, the JMP 9.0 software (SAS Institute Inc., NC, USA) was used. Contingency tables were calculated to study association between HOXB13 staining and clinico-pathological variables, and the Chi-square (Likelihood) test was used to find significant relationships. Kaplan Meier curves were generated for PSA recurrence free survival. The log-Rank test was applied to test the significance of differences between stratified survival functions. Cox proportional hazards regression analysis was performed to test the statistical independence and significance between pathological, molecular, and clinical variables.

## SUPPLEMENTARY TABLES



## Abbreviations

AJCC: American joint committee on cancer
AKT: protein kinase B
AR: androgen receptor
CCND1: cyclin D1
CHD1: chromodomain-helicase DNA binding protein 1
cT: clinical tumor stage
CRISP3: cysteine-rich secretory protein 3
ERG: V-Ets avian erythroblastosis virus E26 oncogene homolog
FISH: fluorescence *in situ* hybridization
FOXP1: forkhead box protein 1
HOXB13: homeobox protein B13
IHC: immunohistochemistry
Ki67LI: Ki67 labeling index
MAP3K7: mitogen-activated protein kinase 7
mTOR: mammalian target of rapamycin
PSA: prostate-specific antigen
PTEN: phosphatase and tensin homolog
pT: pathological tumor stage
pN: pathological lymph node status
TMA: tissue micro array
TMPRSS2: transmembrane protease serine 2.
