# Supplementary material for: HOXB13 overexpression is an independent predictor of early PSA recurrence in prostate cancer treated by radical prostatectomy
Source: Oncotarget. 2015 Mar 23;6(14):12822–34. doi: 10.18632/oncotarget.3431 (PMC4494977; doi:10.18632/oncotarget.3431)
Supplement: Supplementary file 1 [file oncotarget-06-12822-s001.pdf]

SUPPLEMENTARY TABLES

Supplementary Table S1: Pathological and clinical data of the arrayed prostate cancers

	No. of patients (%)	
	Study cohort on TMA	Biochemical relapse among categories
**Follow-up (mo)**		
n	11,665 (93.9%)	2,769 (23.7%)
Mean	48.9	-
Median	36.4	-
**Age (y)**		
≤ 50	334 (2.7%)	81 (24.3%)
51–59	3,061 (24.8%)	705 (23%)
60–69	7,188 (58.2%)	1,610 (22.4%)
≥ 70	1,761 (14.3%)	370 (21%)
**Pretreatment PSA (ng/ml)**		
< 4	1,585 (12.9%)	242 (15.3%)
4–10	7,480 (60.9%)	1,355 (18.1%)
> 10–20	2,412 (19.6%)	737 (30.6%)
> 20	812 (6.6%)	397 (48.9%)
**pT stage (AJCC 2002)**		
pT2	8,187 (66.2%)	1,095 (13.4%)
pT3a	2,660 (21.5%)	817 (30.7%)
pT3b	1,465 (11.8%)	796 (54.3%)
pT4	63 (0.5%)	51 (81%)
**Gleason grade**		
≤ 3 + 3	2,983 (24.1%)	368 (12.3%)
3 + 4	6,945 (56.2%)	1,289 (18.6%)
4 + 3	1,848 (15%)	788 (42.6%)
≥ 4 + 4	584 (4.7%)	311 (53.3%)
**pN stage**		
pN0	6,970 (91%)	1,636 (23.5%)
pN+	693 (9%)	393 (56.7%)
**Surgical margin**		
Negative	9,990 (81.9%)	1,848 (18.5%)
Positive	2,211 (18.1%)	853 (38.6%)

Percentage in the column "Study cohort on TMA" refers to the fraction of samples across each category. Percentage in column "Biochemical relapse among categories" refers to the fraction of samples with biochemical relapse within each parameter in the different categories. Numbers do not always add up to 12,427 (100%) in the different categories because of cases with missing data.

Supplementary Table S2: Association between HOXB13 immunostaining results and prostate cancer phenotype in the ERG negative subset by IHC

	n evaluable	HOXB13 IHC result (%)				*P* value
		negative	weak	moderate	strong	
**All cancers**	5,082	55.9	19	15.7	9.4	
**Tumor stage**						
pT2	3,435	59.6	19.2	13.9	7.3	< 0.0001
pT3a	1,020	50.9	18.3	18.8	12	
pT3b	581	42.7	19.8	20.3	17.2	
pT4	29	48.3	17.2	24.1	10.3	
**Gleason grade**						
≤ 3 + 3	1,185	69	16.6	10	4.3	< 0.0001
3 + 4	2,819	54.6	19.7	16.4	9.3	
4 + 3	782	45.3	20.1	20.3	14.3	
≥ 4 + 4	274	41.6	20.4	19.7	18.2	
**Lymph node metastasis**						
N0	2,896	53.8	19.3	16	10.9	< 0.0001
N+	264	39.4	20.8	22.3	17.4	
**Preop. PSA level (ng/ml)**						
< 4	540	54.1	18.5	18.5	8.9	0.24
4–10	2,999	55.9	20	15	9.2	
> 10–20	1,108	55.2	18	16.6	10.2	
> 20	388	59.8	15.7	14.7	9.8	
**Surgical margin**						
negative	4,084	56	19.5	15.4	9.2	0.18
positive	910	55.8	16.7	16.8	10.7	

Supplementary Table S3: Association between HOXB13 immunostaining results and prostate cancer phenotype in the ERG positive subset by IHC

	n evaluable	HOXB13 IHC result (%)				*P* value
		negative	weak	moderate	strong	
**All cancers**	3,885	36.6	27.4	25.2	10.8	
**Tumor stage**						
pT2	2,333	39.7	28.7	23.2	8.4	< 0.0001
pT3a	1,026	34.2	25.4	26.4	13.9	
pT3b	484	26.7	26.0	31.6	15.7	
pT4	23	30.4	13.0	34.8	21.7	
**Gleason grade**						
≤ 3 + 3	890	49.1	25.7	19.9	5.3	< 0.0001
3 + 4	2,298	34.7	29.2	25.5	10.6	
4 + 3	544	26.5	23.5	31.6	18.4	
≥ 4 + 4	129	24.0	23.3	30.2	22.5	
**Lymph node metastasis**						
N0	2,173	34.7	27.6	25.4	12.2	< 0.0001
N+	222	26.6	22.1	28.8	22.5	
**Preop. PSA level (ng/ml)**						
< 4	530	37.0	28.3	25.3	9.4	0.34
4–10	2,384	36.5	28.1	24.9	10.6	
> 10–20	683	35.0	27.5	25.0	12.4	
> 20	235	40.0	20.4	26.8	12.8	
**Surgical margin**						
negative	3,073	37.3	28.1	24.1	10.5	0.004
positive	744	33.3	24.9	29.8	12.0	
